# Genomic Grade Index (GGI): Feasibility in Routine Practice and Impact on Treatment Decisions in Early Breast Cancer

**DOI:** 10.1371/journal.pone.0066848

**Published:** 2013-08-19

**Authors:** Otto Metzger-Filho, Aurélie Catteau, Stefan Michiels, Marc Buyse, Michail Ignatiadis, Kamal S. Saini, Evandro de Azambuja, Virginie Fasolo, Sihem Naji, Jean Luc Canon, Paul Delrée, Michel Coibion, Pino Cusumano, Veronique Jossa, Jean Pierre Kains, Denis Larsimont, Vincent Richard, Daniel Faverly, Nathalie Cornez, Peter Vuylsteke, Brigitte Vanderschueren, Hélène Peyro-Saint-Paul, Martine Piccart, Christos Sotiriou

**Affiliations:** 1 Institut Jules Bordet, Université Libre de Bruxelles, Brussels, Belgium; 2 Ipsogen, SA, Marseille, France; 3 International Drug Development Institute, Louvain-la-Neuve, Belgium; 4 Grand Hôpital de Charleroi, Charleroi, Belgium; 5 Clinique Saint-Vincent, Rocourt, Belgium; 6 CH St Joseph, Liège, Belgium; 7 Hôpitaux Iris-Sud, Brussels, Belgium; 8 CHU Ambroise Paré, Mons, Belgium; 9 CHU Tivoli, La Louvière, Belgium; 10 Clinique et Maternité Sainte-Elizabeth, Liège, Belgium; 11 RHMS - Clinique Louis Caty, Baudour, Belgium; National University of Ireland Galway, Ireland

## Abstract

**Purpose:**

Genomic Grade Index (GGI) is a 97-gene signature that improves histologic grade (HG) classification in invasive breast carcinoma. In this prospective study we sought to evaluate the feasibility of performing GGI in routine clinical practice and its impact on treatment recommendations.

**Methods:**

Patients with pT1pT2 or operable pT3, N0-3 invasive breast carcinoma were recruited from 8 centers in Belgium. Fresh surgical samples were sent at room temperature in the Map*Quant* Dx™ PathKit for centralized genomic analysis. Genomic profiles were determined using Affymetrix U133 Plus 2.0 and GGI calculated using the Map*Quant* Dx® protocol, which defines tumors as low or high Genomic Grade (GG-1 and GG-3 respectively).

**Results:**

180 pts were recruited and 155 were eligible. The Map*Quant* test was performed in 142 cases and GGI was obtained in 78% of cases (n=111). Reasons for failures were 15 samples with <30% of invasive tumor cells (11%), 15 with insufficient RNA quality (10%), and 1 failed hybridization (<1%). For tumors with an available representative sample (≥ 30% inv. tumor cells) (n=127), the success rate was 87.5%. GGI reclassified 69% of the 54 HG2 tumors as GG-1 (54%) or GG-3 (46%). Changes in treatment recommendations occurred mainly in the subset of HG2 tumors reclassified into GG-3, with increased use of chemotherapy in this subset.

**Conclusion:**

The use of GGI is feasible in routine clinical practice and impacts treatment decisions in early-stage breast cancer.

**Trial Registration:**

ClinicalTrials.gov NCT01916837, http://clinicaltrials.gov/ct2/show/NCT01916837

## Introduction

Integrating the results of genomic tests with classic clinico-pathologic variables to make treatment decisions represents a major advance in early breast cancer [1]. Several gene expression signatures have been developed with the aim of refining the prognosis of patients diagnosed with this disease [1]. Retrospective analyses have demonstrated the ability of genomic signatures to classify patients into risk categories, thereby adding prognostic value to classic clinico-pathologic variables [2–4]. Moreover, such signatures provide predictive information, identifying the subgroups of patients most likely to benefit from therapeutic interventions such as chemotherapy [5,6]. The value of these signatures for use in clinical practice, however, is still under investigation in three prospective phase III clinical trials [7–9].

A major concern is the feasibility of incorporating genomic signatures into routine clinical procedures. As for any practice-changing technology, a careful evaluation of the logistics associated with its use is needed in order to ensure future applicability. This is particularly true for molecular assays requiring fresh or frozen tumor samples, which add complexity to routine surgical and pathology procedures.

GGI is a 97-gene microarray signature developed to refine histologic grade (HG) assessment, mainly capturing the expression of proliferation genes. In a seminal study, GGI was shown to improve the prognostic value of classic Elston-Ellis histologic grading in patients diagnosed with early breast cancer [3,10]. GGI is particularly useful for segregating HG2 tumors into low (GG-1) or high (GG-3) genomic grade with clinical behavior similar to HG1 and HG3 respectively, and has been recommended as a complement to histologic grade by the 2009 St Gallen consensus [11]. Further analyses have demonstrated the ability of GGI to categorize estrogen receptor (ER)-positive tumors into two subtypes with distinct clinical outcomes based on the differences in their proliferation rates [12,13].

Evidence for GGI predictive value was provided in a retrospective analysis of patients treated with neoadjuvant chemotherapy [14]. High GGI was associated with increased sensitivity to neoadjuvant anthracycline and taxane-based chemotherapy in both ER-positive and ER-negative breast cancer subsets. This finding was confirmed in a recent pooled analysis of almost 1000 patients [15].

The present study prospectively evaluated both the feasibility of implementing GGI in eight community-based hospitals and the impact of GGI results on treatment decisions.

## Patients and Methods

All patients provided written informed consent prior to enrolment. The protocol was approved by the Jules Bordet Institute’s central ethics committee, and local ethics committees reviewed the protocol at each participating institution. The study was registered with the European Community’s Drug Regulatory Authorities Clinical Trial System (EudraCT 2009-015521-36). The protocol for this trial and supporting CONSORT checklist are available as supporting information; see Checklist S1 and Protocol S1. Eligible patients included women with histologically confirmed pT1-pT2 or pT3 operable primary invasive breast cancer with no more than three positive nodes (defined as lymph node metastases >2mm) and absence of distant metastasis. Patients with multifocal tumors were eligible, provided that a sample from each tumor was taken for the GGI test. Patients with in situ disease were eligible if invasive carcinoma was present. Patients with previous invasive cancer within the past five years were ineligible, except for patients treated adequately for carcinoma in situ of the cervix or non-melanoma skin cancer.

### Objectives

The primary objective of this study was to evaluate the feasibility of implementing GGI in community hospitals in Belgium for breast cancer patients with node negative and 1-3 node positive early breast cancer. As defined in the protocol, GGI would be considered a feasible genomic test if results were obtained in > 70% of evaluated patients. The secondary objective was to evaluate the impact of GGI on adjuvant treatment decisions for patients with early breast cancer. This was done by comparing physicians’ treatment recommendations before having knowledge of the GGI test results to recommendations with a hypothetical GG-1 and GG-3 result and to the treatment ultimately administered after discussion with the patient.

### Participating centers and study material

Participating hospitals were required to have multidisciplinary breast cancer care structures and a tumor board in place and at least one dedicated physician (surgeon, pathologist, or medical oncologist) as a local coordinator. Eight Belgian hospitals participated in the study, and the logistics of the study was coordinated by the Jules Bordet Institute. At the initiation of the study, an instructional meeting was organized with all participating hospitals to discuss the logistics and their integration into routine clinical practice. An instructional video in which the full procedure – from sampling to shipping was demonstrated by an experienced pathologist was also provided to each pathologist involved in the study. Ready-to-use sample collection Map*Quant* Dx™ PathKits (Ipsogen) containing all necessary material for tumor sampling were provided to collect and ship tumors.

### Tumor sampling and shipment

Fresh tumor specimens were sampled prior to adding any fixative and within one hour of surgery. Investigators were instructed to select a fragment from the periphery of the tumor where the tumor is the most proliferative, and to avoid its center, which is often necrotic and fibrotic. The minimum size required was 3mm diameter. Alternatively, frozen samples could be provided. In this case, 10x20μm thick sections per tumor sample were required. Samples (fresh or frozen) were immersed immediately into the RNAlater^®^ preserving solution (Ambion Inc., Austin, TX, USA) and kept at 4°C (up to 3 weeks) or at room temperature (up to one week) until shipped.

One Hematoxylin and Eosin (H&E) slide representative of the tumor sample was also required in order to check the content of invasive tumor cells. The H&E slide was prepared from a section adjacent to the sample taken for the test. The Map*Quant* Dx PathKits containing single tumor samples, a representative H&E slide, and a sample report form (SRF) (for pathology laboratory information, study number, sampling procedure, storage condition, sampling date, shipping date, and kit-use checking controls) were sent at room temperature to a central laboratory (DNA Vision SA, Charleroi, Belgium), where the microarray analyses were performed.

### Microarray analysis and GGI determination

Central pathology review was performed on all tumor samples received at the central laboratory. The H&E slides received had to contain at least 30% of invasive tumor cells for further analysis. RNA was extracted and purified with the RNeasy MiniKit® according to the manufacturer’s instructions (Qiagen). RNA was quantified and qualitatively assessed on triplicate samples on a NanoDrop® ND-1000 UV–Vis Spectrophotometer (NanoDrop Technologies, Wilmington, DE, USA) and Agilent 2100 Bioanalyzer (Agilent Technology, Santa Clara, CA). Samples with ≥ 100 ng and a RIN ≥ 7 were qualified for microarray analyses performed on Affymetrix U133 Plus 2.0 arrays according to Map*Quant* Dx conditions (GEO accession number GSE43365). GGI was computed using the Ipsogen Map*Quant* Dx protocol (Table S1), which defines tumors as low GGI (GG-1) or high GGI (GG-3), based on GGI values. To ensure the robustness and accuracy of genomic grading as per the Map*Quant* protocol, when the GGI value of a sample could not be ascribed with a probability ≥ 75% to either GG-1 or GG-3, it was defined as equivocal (Eq).

### Study flow

At each participating center, a Case Report Form 1 (CRF section 1) was filled-in after surgery and pathological assessment. The records described the following: patient characteristics; definitive tumor histology; histologic grade (assessed according to Elston-Ellis recommendations); ER, progesterone receptor (PR) and HER2 status; tumor availability for the genomic test; and systemic adjuvant treatment recommendations by the treating physicians. Treatment recommendations were based on 1) patient and tumor characteristics, and 2) patient and tumor characteristics plus hypothetical GGI results (GG-1 or GG-3), each center using its usual routine decision algorithm. The actual GGI results were provided to participating centers only after receipt of the completed CRF section 1. Once this information was received, the physicians discussed treatment recommendations with their patients. The final treatment prescribed was then reported on CRF section 2, which was sent back to the coordinating center.

### Statistical analysis

To test the success rate of reporting GGI, a sample size of 137 was calculated to have a power of 85% at a one-sided significance level of 5% using an empirical estimate of variance (null hypothesis H0: p = 0.70 vs. alternative hypothesis HA: p > 0.70) when the true success rate is equal to the prespecified value of 0.80 under the alternative (using an empirical estimate of variance). The original critical value from which the null hypothesis could be rejected was 106 successes out of 137. Descriptive analyses were performed to classify patients according to clinico-pathologic characteristics, HG and GGI, initial treatment recommendations, treatment recommendations after GGI results, and final treatment prescribed to patients. Concordance between HG and GGI was defined as the proportion of patients classified as HG1/GG-1 and HG3/GG-3. Statistical analysis was performed using SAS version 9.2

## Results

### Patient demographics

Between February and December 2010, 180 patients were enrolled across eight participating hospitals in Belgium. Following initial breast cancer surgery, a total of 25 patients were excluded because of the presence of more than three positive nodes (n = 22) or non-invasive tumor (n = 3).

The clinico-pathologic characteristics of the 155 eligible patients are summarized in table 1 and outlined here: the median age was 38 years (range 31-90); most patients were considered to be in either perfect health (43%) or to have only minor problems (33%); the median tumor diameter was 1.6 cm (range 0.6 cm to 5.5 cm); most patients had T1 tumors (68%) and node-negative disease (79%); and histological grade classified 38 tumors as HG1 (24%), 77 as HG2 (50%) and 40 as HG3 (26%).

**Table 1:** Patient and tumor characteristics (n = 155).

Age at diagnosis	
Median [min-max]	38 [31-90]
	**n (%)**
Menopausal status	
Premenopausal	43 (28%)
Postmenopausal	94 (61%)
Unknown	18 (11%)
Comorbidities	
Perfect health	66 (43%)
Minor problems	51 (33%)
Average for age	21 (13%)
Major problems	17 (11%)
Tumor size (cm)	
Median [Min-Max]	1.6 [0.6-5.5]
T1	105 (68%)
T2	49 (31%)
T3	1 (1%)
Nodal Status	
Negative	122 (79%)
1-3 positive	33 (21%)
ER/PR Status	
ER pos / PR pos	115 (74%)
ER pos / PR neg	20 (13%)
ER neg / PR neg	20 (13%)
Histologic Grade	
HG1	38 (24%)
HG2	77 (50%)
HG3	40 (26%)
Unknown	1 (2%)
HER2 Status	
positive	15 (10%)
negative	138 (89%)
unknown	2 (1%)

**Abbreviations:** ER/PR, estrogen receptor/ progesterone receptor.

### Procedures

**Figure 1:**
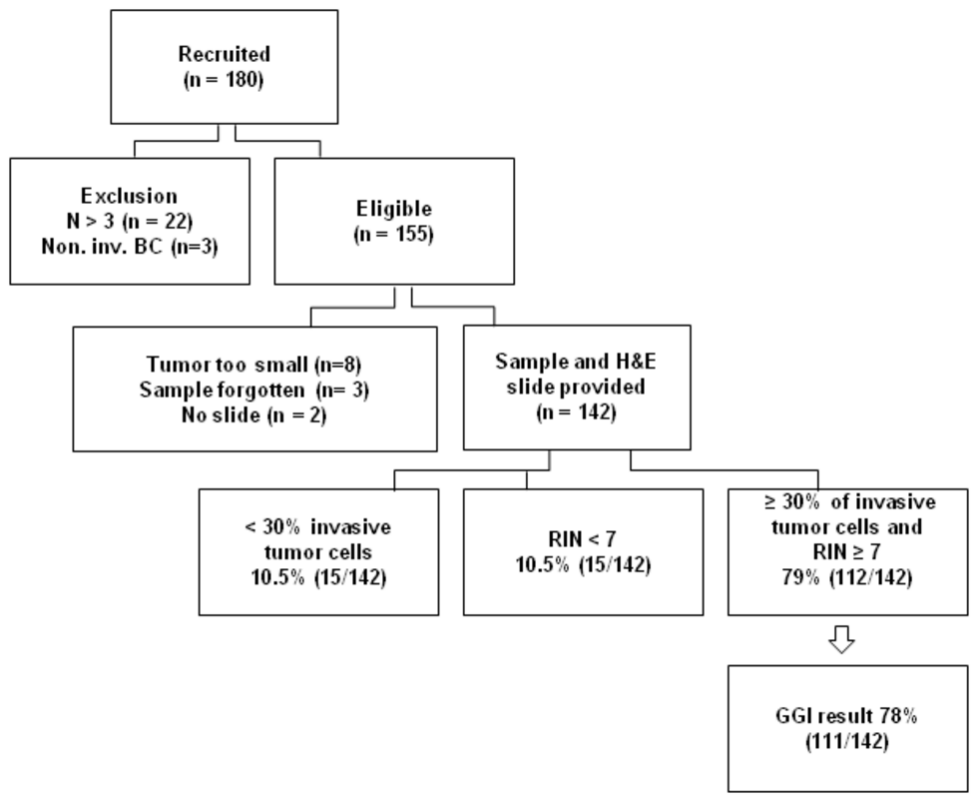
Flow diagram of enrolled patients. **Abbreviations**: inv, invasive; N, nodal status.

A flow diagram of recruited patients and selected tumors is depicted in Figure 1. Out of 155 eligible patients, a total of 13 patients could not be retained for further genomic analysis (8 tumors estimated too small for sampling; no sample available for 3 patients; and 2 tumor samples sent without H&E slide). The median tumor size of the 8 tumors that were not sampled was 0.8 cm (range 0.6 cm to 1 cm). The 142 evaluated patients for whom a tumor sample and representative H&E slide were provided were thus included for the primary endpoint analysis. However, 15 tumors were excluded after central pathology assessment because of insufficient invasive tumor cell content (<30%), and an additional 15 tumor samples failed the RNA qualification. Out of the 112 remaining samples, hybridization was successful for 111 (99%).

With 111 successes out of 142 (78.2%, with a lower bound of the exact one-sided 95% confidence interval provided by 71.7%) the null hypothesis that the true probability is equal to 70% is rejected with a one-sided p-value of 0.017. In the subset of patients with ≥30% invasive tumor cells in the representative slide, GGI was obtained with a success rate of 87.5% (111/127).

### Comparison between histologic grade and genomic grade classification

Cross-tabulations of histologic and genomic grade for the 111 patients with a GGI result are shown in table 2. A total of 21 (19%) tumors were classified as HG1, 54 (49%) as HG2 and 36 (32%) as HG3. GGI classified 39 (35%) tumors as GG-1, 48 (43%) as GG-3, and 24 (22%) as equivocal. Within the HG2 subgroup representing 49% of the cohort, GGI reclassified 69% of the cases (n=37) into GG-1 (n =20, 54%) and GG-3 (n = 17, 46%), respectively. The overall concordance between HG1 (n=15)/GG-1 (n=19) and HG3 (n=30)/GG-3 (n=31) was 90%. GG-Equivocal results were not included in the overall concordance estimate as it is non-informative for treatment decision.

**Table 2:** Genomic Grade Index classification performance.

**GGI**	**Histological Grade**			
	**HG1**	**HG2**	**HG3**	**Total**
**GG-1 (n)**	15	20	4	39 (35%)
**Equivocal (n)**	5	17	2	24 (22%)
**GG-3 (n)**	1	17	30	48 (43%)
**Total (n, %)**	21 (19%)	54 (49%)	36 (32%)	111

**Abbreviations:** GGI, genomic grade index; HG, histologic grade

### Impact of genomic grade on treatment decisions

We first analyzed the potential impact of GGI on treatment decisions in the subgroup of patients with ER-positive/HER2-negative breast cancer where tumor grade is one of the key factors driving chemotherapy decision. Out of 155 eligible patients (described in Figure 1), 126 were classified as ER-positive and HER2-negative. Among these, 38 (30%) tumors were classified as HG1, 67 (53%) as HG2 and 21 (17%) as HG3. The physicians’ recommendations for adjuvant chemotherapy based on clinico-pathologic features were compared to their recommendations for chemotherapy based on clinico-pathologic features plus hypothetical GGI results (either GG-1 or GG-3). Physicians recommended chemotherapy to 33% of the patients based on clinico-pathologic features. Adding a hypothetical GG-1 to the clinico-pathologic features decreased the chemotherapy indications to 25%, while a hypothetical GG-3 result increased them to 54% as detailed in table 3. A similar analysis of the HG2 subgroup (n = 68) revealed that physicians recommended chemotherapy to 31% of patients based on clinico-pathologic features, to 24% based on clinico-pathologic features plus hypothetical GG-1 and to 59% when based on clinico-pathologic features plus hypothetical GG-3.

**Table 3:** Treatment recommendation based on hypothetical GGI results for patients diagnosed with ER-positive and HER2-negative breast cancer.

**Treatment recommendation**	**Clinico-pathologic characteristics (n, %)**	**Hypothetical GG-1 (n, %)**	**Hypothetical GG-3 (n, %)**
**CHT**	42 (33%)	32 (25%)	68 (54%)
**HT**	84 (67%)	94 (75%)	58 (46%)

**Abbreviations:** CHT, chemotherapy plus hormonotherapy; GGI, genomic grade index; HT, hormonotherapy; tx, treatment.

**Note:** Hypothetical GG-1 and GG-3 refer to recommended tx based on clinico-pathologic characteristics and assuming that patients were hypothetically classified as GG-1 and GG-3, respectively.

**Figure 2:**
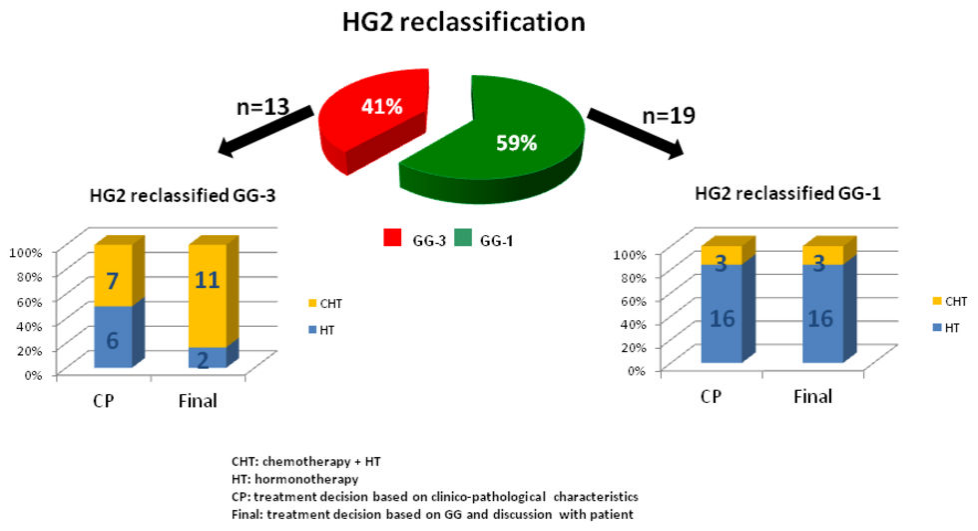
Impact of GGI reclassification on treatment decisions in ER+ and HER2-negative and HG2 early-stage breast cancer. **Abbreviations**: CP, treatment decision based on clinico-pathologic characteristics; CHT, chemotherapy plus hormonotherapy; HT, hormonotherapy; final, final treatment decision based on GG results and discussion with patient.

As a second step, we evaluated the impact of GGI on final treatment decisions for patients diagnosed with HG2/ER-positive/HER2-negative breast cancers reclassified either as GG-1 or GG-3. Out of 111 patients for which the GGI test was available, 32 patients were diagnosed with HG2/ER-positive/HER2-negative breast cancer and reclassified as GG-1 (n=19) or GG-3 (n=13) as shown in Figure 2. One of the 3 HG2/GG-1 patients initially proposed for chemo-hormonotherapy (CHT) was finally treated with hormonotherapy only, while one patient initially recommended for hormonotherapy finally received CHT. By contrast, while CHT was initially recommended for 7 patients out of the 13 HG2/GG-3 (54%), 11 received CHT (85%). No changes in treatment recommendation occurred in the subset of patients with ER-positive breast cancer with HG1 or HG3 and reclassified into GGI equivocal.

## Discussion

The results of this prospective study demonstrate that measuring GGI is feasible in routine clinical practice. Collecting fresh tumor samples, shipping them to a central laboratory for gene expression profiling, and obtaining GGI results was achieved successfully in 78% of evaluated cases. In the subset of patients with adequate tumor sampling (representative H&E slide provided with ≥30% invasive tumor cells) GGI was feasible in 87.5% of cases. Overall concordance for HG1/GG-1 and HG3/GG-3 was 90%, and GGI reclassified 69% HG2 tumors, thereby providing additional information in cases where histologic grade is uninformative. This HG2 reclassification rate was identical to the one observed in a recent study in which Map*Quant* GGI was performed on a retrospective series of 163 early breast cancer samples [16].

Previous studies evaluating the feasibility of integrating genomic microarray technologies requiring fresh or frozen tumor tissue into the early treatment of breast cancer have reported similar success rates [17,18]. The 70-gene prognostic signature (MammaPrint^TM^) was prospectively evaluated in a study involving 16 community-based hospitals in the Netherlands, and the results were successfully provided for 73% of eligible patients (427 of 585) [18]. In a subsequent pilot study, MammaPrint^TM^ achieved a success rate of 72% (46 out of 64) [17], and reinforced the conduct of the completely enrolled 6600-patient phase III study MINDACT (Microarray in Node-negative and 1-3 node-positive disease may Avoid ChemoTherapy), evaluating whether there is benefit to combining MammaPrint^TM^ with clinic-pathologic features for treatment decision-making in early breast cancer [7].

When compared to previous reports [17,18], it is clear that our study has been conducted in an era in which genomics are being increasingly implemented in clinical practice. This is especially true in the United States, where the 21-gene recurrence score (Onco*type* DX™) is endorsed by the American Society of Clinical Oncology as a useful tool to predict prognosis and risk of recurrence in patients with node-negative, ER-positive breast cancer, and reimbursed [19]. The fact that genomic tests can improve our understanding of breast cancer biology and that such genomic tests are currently not reimbursed in Europe probably influenced investigators to rapidly enroll patients in our study. The 180 early-stage breast cancer patients were enrolled within only 10 months in 8 community-based hospitals. The oncology-tertiary center, Jules Bordet Institute, coordinated the study without enrolling patients. This indicates that genomic tests can easily be implemented in routine practice.

In regard to treatment plans, participating physicians used the GGI results in combination with their usual treatment algorithm, i.e., without pre-specified treatment guidance. Tumor boards were asked to establish their treatment strategy based on their usual clinico-pathologic features, and then using the hypothetical and final GGI results. The final treatment decision was ultimately made after discussion with each patient. The exercise based on hypothetical GGI results performed before informing investigators of the final results demonstrated that physicians are willing to increase chemotherapy indications for those classified as GG-3 and to decrease chemotherapy indications for those classified GG-1

Upon receiving the final GG results, physicians changed their treatment recommendations mostly for patients with discordant GGI and HG results, therefore mainly for the HG2 cases, which represents the optimal target population for the Genomic Grade Index. However, the small numbers of HG2 cases in our study, which primary objective was to assess feasibility of the GGI in the routine of a breast cancer care centre, limits our ability to draw definitive conclusions. The difference between the hypothetical scenarios and the final treatment decisions is likely to reflect patient preferences. Similar results were observed in a prospective study evaluating the impact of Onco*type* DX™ on the treatment selections of medical oncologists and patients [20]. Out of a total of 89 early stage breast cancer cases, medical oncologists changed their pre- and post-test treatment recommendations from chemotherapy plus hormonotherapy to hormonotherapy in 22.5% of cases, but when patient preferences were taken into consideration, the same changes occurred in only 10.1% of the cases.

In conclusion, the present prospective study demonstrates that determining GGI is achievable in community hospitals, which reinforces the feasibility of incorporating genomic technologies into routine clinical practice. In addition, integrating GGI into clinico-pathologic risk assessment may guide physicians and patients towards better individualization of treatment decisions for early stage breast cancer. However, the clinical usefulness of genomic tools is still uncertain and is under evaluation in prospective randomized clinical trials.

## Supporting Information

Table S1 **Map*Quant* Dx Protocol.**
(DOCX)

Checklist S1 **Remark checklist.**
(DOCX)

Protocol S1 **Study Protocol - MapQuant Dx Genomic Grade: feasibility in routine practice and impact of tumor grade quantification on treatment decision-making in early breast cancer patients.**
(PDF)

Text S1 **Map*Quant* Dx Process.**
(PDF)

Text S2 **Central Ethics Committee Approval.**
(PDF)

## References

[1] Sotiriou C, Pusztai L (2009) Gene-expression signatures in breast cancer. N Engl J Med 360: 790-800. doi:10.1056/NEJMra0801289. PubMed: 19228622. 19228622 10.1056/NEJMra0801289

[2] van de Vijver MJ, He YD, van’t Veer LJ, Dai H, Hart AA et al. (2002) A gene-expression signature as a predictor of survival in breast cancer. N Engl J Med 347: 1999-2009. doi:10.1056/NEJMoa021967. PubMed: 12490681. 12490681 10.1056/NEJMoa021967

[3] Sotiriou C, Wirapati P, Loi S, Harris A, Fox S et al. (2006) Gene expression profiling in breast cancer: understanding the molecular basis of histologic grade to improve prognosis. J Natl Cancer Inst 98: 262-272. doi:10.1093/jnci/djj052. PubMed: 16478745. 16478745 10.1093/jnci/djj052

[4] Paik S, Shak S, Tang G, Kim C, Baker J et al. (2004) A multigene assay to predict recurrence of tamoxifen-treated, node-negative breast cancer. N Engl J Med 351: 2817-2826. doi:10.1056/NEJMoa041588. PubMed: 15591335. 15591335 10.1056/NEJMoa041588

[5] Albain KS, Barlow WE, Shak S, Hortobagyi GN, Livingston RB et al. (2010) Prognostic and predictive value of the 21-gene recurrence score assay in postmenopausal women with node-positive, oestrogen-receptor-positive breast cancer on chemotherapy: a retrospective analysis of a randomised trial. Lancet Oncol 11: 55-65. doi:10.1016/S1470-2045(09)70314-6. PubMed: 20005174. 20005174 10.1016/S1470-2045(09)70314-6 PMC3058239

[6] Paik S, Tang G, Shak S, Kim C, Baker J et al. (2006) Gene expression and benefit of chemotherapy in women with node-negative, estrogen receptor-positive breast cancer. J Clin Oncol 24: 3726-3734. doi:10.1200/JCO.2005.04.7985. PubMed: 16720680. 16720680 10.1200/JCO.2005.04.7985

[7] Cardoso F, Van’t Veer L, Rutgers E, Loi S, Mook S et al. (2008) Clinical application of the 70-gene profile: the MINDACT trial. J Clin Oncol 26: 729-735. doi:10.1200/JCO.2007.14.3222. PubMed: 18258980. 18258980 10.1200/JCO.2007.14.3222

[8] Sparano JA, Paik S (2008) Development of the 21-gene assay and its application in clinical practice and clinical trials. J Clin Oncol 26: 721-728. doi:10.1200/JCO.2007.15.1068. PubMed: 18258979. 18258979 10.1200/JCO.2007.15.1068

[9] Hayes DF (2012) Targeting Adjuvant Chemotherapy: A Good Idea That Needs to Be Proven! J Clin Oncol, 30: 1264–7. PubMed: 22355050. 22355050 10.1200/JCO.2011.38.4529

[10] Filho OM, Ignatiadis M, Sotiriou C (2010) Genomic Grade Index: An important tool for assessing breast cancer tumor grade and prognosis. Crit Rev Oncol/Hematol 77: 20-29. 10.1016/j.critrevonc.2010.01.011 20138540

[11] Goldhirsch A, Ingle JN, Gelber RD, Coates AS, Thürlimann B et al. (2009) Thresholds for therapies: highlights of the St Gallen International Expert Consensus on the primary therapy of early breast cancer 2009. Ann Oncol 20: 1319-1329. doi:10.1093/annonc/mdp322. PubMed: 19535820. 19535820 10.1093/annonc/mdp322 PMC2720818

[12] Loi S, Haibe-Kains B, Desmedt C, Lallemand F, Tutt AM et al. (2007) Definition of clinically distinct molecular subtypes in estrogen receptor-positive breast carcinomas through genomic grade. J Clin Oncol 25: 1239-1246. doi:10.1200/JCO.2006.07.1522. PubMed: 17401012. 17401012 10.1200/JCO.2006.07.1522

[13] Metzger-Filho O, Michiels S, Bertucci F, Catteau A, Salgado R et al. (2012) Genomic grade adds prognostic value in invasive lobular carcinoma. Ann Oncol, 24: 377–84. PubMed: 23028037. 23028037 10.1093/annonc/mds280

[14] Liedtke C, Hatzis C, Symmans WF, Desmedt C, Haibe-Kains B et al. (2009) Genomic grade index is associated with response to chemotherapy in patients with breast cancer. J Clin Oncol 27: 3185-3191. doi:10.1200/JCO.2008.18.5934. PubMed: 19364972. 19364972 10.1200/JCO.2008.18.5934 PMC2716940

[15] Ignatiadis M, Singhal SK, Desmedt C, Haibe-Kains B, Criscitiello C et al. (2012) Gene modules and response to neoadjuvant chemotherapy in breast cancer subtypes: a pooled analysis. J Clin Oncol 30: 1996-2004. doi:10.1200/JCO.2011.39.5624. PubMed: 22508827. 22508827 10.1200/JCO.2011.39.5624

[16] Reyal F, Bollet MA, Caly M, Gentien D, Carpentier S et al. (2012) Respective prognostic value of genomic grade and histological proliferation markers in early stage (pN0) breast carcinoma. PLOS ONE 7: e35184. doi:10.1371/journal.pone.0035184. PubMed: 22529987. 22529987 10.1371/journal.pone.0035184 PMC3329444

[17] Mook S, Bonnefoi H, Pruneri G, Larsimont D, Jaskiewicz J et al. (2009) Daily clinical practice of fresh tumour tissue freezing and gene expression profiling; logistics pilot study preceding the MINDACT trial. Eur J Cancer 45: 1201-1208. doi:10.1016/j.ejca.2009.01.004. PubMed: 19232484. 19232484 10.1016/j.ejca.2009.01.004

[18] Bueno-de-Mesquita JM, van Harten WH, Retel VP, van’t Veer LJ, van Dam FS et al. (2007) Use of 70-gene signature to predict prognosis of patients with node-negative breast cancer: a prospective community-based feasibility study (RASTER). Lancet Oncol 8: 1079-1087. doi:10.1016/S1470-2045(07)70346-7. PubMed: 18042430. 18042430 10.1016/S1470-2045(07)70346-7

[19] Harris L, Fritsche H, Mennel R, Norton L, Ravdin P et al. (2007) American Society of Clinical Oncology 2007 update of recommendations for the use of tumor markers in breast cancer. J Clin Oncol 25: 5287-5312. 17954709 10.1200/JCO.2007.14.2364

[20] Lo SS, Mumby PB, Norton J, Rychlik K, Smerage J et al. (2010) Prospective multicenter study of the impact of the 21-gene recurrence score assay on medical oncologist and patient adjuvant breast cancer treatment selection. J Clin Oncol 28: 1671-1676. doi:10.1200/JCO.2008.20.2119. PubMed: 20065191. 20065191 10.1200/JCO.2008.20.2119

