# Supplementary material for: Genomic Grade Index (GGI): Feasibility in Routine Practice and Impact on Treatment Decisions in Early Breast Cancer
Source: PLoS One. 2013 Aug 19;8(8):e66848. doi: 10.1371/journal.pone.0066848 (PMC3747186; doi:10.1371/journal.pone.0066848)
Supplement: Table S1 — MapQuant Dx Protocol. (DOCX) [file pone.0066848.s001.docx]

### Genomic Grade Index (GGI): Feasibility in routine practice and impact on treatment decisions in early breast cancer

**Table S1**

| Map*Quant* Dx protocolQuality Control Quality of Affymetrix chips data was assessed with the Map*Quant* Dx protocol which combines several Affymetrix quality controls steps (using average background and average noise values, Scale factor and Percent Present, GAPDH and β-actin, and degradation slope). Calibration Each microarray sample was calibrated against an internal reference set of 191 breast cancer profiles (191 U133 Plus 2.0 arrays from Ipsogen’s Affymetrix platform) using RMADx, which is based on the standard Robust Multi-Array (RMA) algorithm. RMA corrects for background values, performs normalization (quantile normalization) and summarizes probe signals into probeset signals (median polish). In RMADx the quantile normalization and probe summarization with median-polish use the fixed internal reference set to calibrate any new array. Computation of Genomic Grade Index (GGI) Continuous GGI values were calculated by the weighted average formula of 128 probe sets (corresponding to 97 genes) as in Sotiriou et al (3). GGI values were scaled against a retrospective scaling set of arrays so that the average GGI of histological grade 1 tumors is -1 and that of grade 3 tumors is +1. The fixed retrospective scaling set consists of 112 arrays from 112 ER-positive breast cancer samples (53 HG1 and 59 HG3 as measured by reference pathology centers as per Elston and Ellis) profiled on 3 Affymetrix platforms that have been demonstrated to be have low between-laboratory variability in GGI values obtained with same RNA as compared to the reference Ipsogen Affymetrix platform. Determination of GG1, GG3 or equivocal cases Cutoffs to classify tumor samples into the GG1, GG3 categories or as “equivocal” have been determined on the fixed retrospective scaling set as follows. The genomic grade distribution of HG1 patients and HG3 patients was previously found to be approximately normally distributed in a large patient series (4). The genomic grade distribution in the scaling set HG1 and HG3 populations could thus be fitted to normal distributions and probabilities curves calculated. When a new sample has less than 75% probability to be classified as either GG1 or GG3, it is considered “equivocal”. The cut-offs are given by - 0.34 and + 0.34.  **References**  1. Sotiriou C, Wirapati P, Loi S, et al: Gene expression profiling in breast cancer: understanding the molecular basis of histologic grade to improve prognosis. J Natl Cancer Inst 98:262-72, 2006  2. Wirapati P, Sotiriou C, Kunkel S, et al: Meta-analysis of gene expression profiles in breast cancer: toward a unified understanding of breast cancer subtyping and prognosis signatures. Breast Cancer Res 10:R65, 2008  3. Ignatiadis M, Singhal SK, Desmedt C, et al: Gene Modules and Response to Neoadjuvant Chemotherapy in Breast Cancer Subtypes: A Pooled Analysis. J Clin Oncol, 2012 Epub 2012 Apr 16  4. Fraley C and Raftery A: Model-based clustering, discriminant analysis, and density estimation. Journal of the American Statistical Association, 97: 611-631, 2002. |
| --- |
