# Supplementary material for: Genomic Grade Index (GGI): Feasibility in Routine Practice and Impact on Treatment Decisions in Early Breast Cancer
Source: PLoS One. 2013 Aug 19;8(8):e66848. doi: 10.1371/journal.pone.0066848 (PMC3747186; doi:10.1371/journal.pone.0066848)
Supplement: Protocol S1 — Study Protocol - MapQuant Dx Genomic Grade: feasibility in routine practice and impact of tumor grade quantification on treatment decision-making in early breast cancer patients. (PDF) [file pone.0066848.s003.pdf]

GGI: FEASIBILITY and IMPACT on TREATMENT DECISION in INVASIVE BREAST CANCER

Title of Study:

MapQuant Dx™ Genomic Grade: feasibility in routine practice and impact of tumor grade quantification on treatment decision-making in early breast cancer patients.

Principal Investigators: Christos Sotiriou MD, PhD; Martine Piccart MD, PhD (Institut Jules Bordet, Brussels, Belgium)

Research Fellow: Otto Metzger MD (Institut Jules Bordet, Brussels, Belgium)

Statistical Analysis: Marc Buyse, IDDI, Brussels, Belgium

1. Study Rational

Breast cancer is the leading cause of death in women between the ages of 40 and 79 years, and the second leading cause of cancer death for women of all ages. The American National Cancer Institute estimates that approximately 2.4 million women with a history of breast cancer were alive in 2004 [1].

In the last 20 years mortality due to breast cancer has declined largely due to improved mammographic screening, but also in part due to the use of adjuvant systemic therapy. It is fundamental, however, that treatment decisions are made to avoid overtreatment and under treatment [2].

Decisions related to the use of adjuvant systemic therapies have relied on traditional clinico-histologic features, models developed to assist physicians such as Adjuvant Online, and recently on the use of molecular tools. However few, if any, of these newer tools have been incorporated into the traditional algorithms used to assist oncologists in estimating the absolute benefit of chemotherapy for an individual patient and supporting treatment decision.

One of the key prognostic factors used in these algorithms is tumor grade, assessed today by histologic methods. However its reproducibility is affected by the variety of approaches used and by the inherently subjective nature of these methods. In other to better quantify tumor grading, Elston and Ellis designed a modification of the Bloom and Richard grading system [13].

The Elston-Ellis system is based on semi-quantitative evaluation of three morphologic features (percentage of tubule formation, degree of nuclear pleomorphism and accurate mitotic count in a defined area). Three grade categories are given using a numerical score system, where the overall grade is derived summing-up the individual scores. In a study of 1831 patients, those with histologic grade I tumors had better survival than those with grade II and III tumors ($p<0.0001$), leading to the American College of Pathologists recommendation of tumor grade as an important prognostic factor in breast cancer. [14]. The latest Breast Task of the American Joint Committee on Cancer evaluated the possibility of adding the tumor grading in the TNM system. In their recommendation, they stated that adding tumor grade to large tumors (T3 and T4) would not affect clinical decision as these large tumors are already treated with chemotherapy quite systematically. However, it would be expected to add valuable information for the node negative T1 and T2 tumors, but the interaction between tumor size and histologic grade, and relationship to outcome, remain poorly understood [15]. From a technical perspective, analysis of tumor grade in many series show that about half of breast cancer patients are assigned to grade 1 or 3 status (with a low or high risk of recurrence, respectively) and a substantial percentage of tumors are classified as histologic grade 2 (30-60%), making the grading uninformative in about half of the patients [12]. Although there is an assumption that the variability in tumor grade assessment could be reduced by increasing the number of pathologists, it was not seen in the context of a clinical trial [16]. Discordance rates ranging from 35% to 41% were observed when two pathologists with experience in breast cancer pathology evaluated tumor grade in the same group of patients. The overall agreement of three pathologists assessing tumor grade in the same subset of patients was even lower (43%).

The gene expression profiling studies were fundamental for a better understanding of the molecular heterogeneity of breast cancer [3-6]. Four main classes of breast cancer have been distinguished namely, basal like breast cancer, corresponding to ER-negative, PR-negative and HER2-negative (triple negative); luminal A cancers, mostly ER positive and histologically low

grade; luminal B, which are also ER positive and are often high grade; and HER2 positive breast cancer, which show amplification and high expression of the ERBB2 gene [7].

Considering low and high grade tumors as low and high proliferative tumors, respectively, the gene profiling studies helped to understand the importance of tumor grade as a prognostic marker. In a meta-analysis of publicly available breast cancer gene expression and clinical data, proliferation genes have been identified as a common driving force behind the performance of the prognostic signatures studied [8].

There is increasing evidence that the benefit of chemotherapy for the group of ER positive breast cancer is not the same as for ER negative disease. The International Breast Cancer Study Group (IBCSG) detected larger chemotherapy benefit in ER negative or low ER invasive cancer compared to ER-intermediate or ER-rich disease in both the IBCSG Trial IX and IBCSG VII [10]. Also the benefit of adding taxanes to doxorubicin based protocols is more impressive in HR-negative disease with a modest treatment effect in HR-positive disease [11]. Overall, adjuvant chemotherapy has globally decreased breast cancer mortality, but the majority of women are still treated unnecessarily to benefit a few. The Oxford Overview analyses showed a significant proportion of long term survivors even among untreated patients [9]. It is therefore crucial to precisely identify the group of patients susceptible to get the maximum benefit from adjuvant chemotherapy, while sparing unnecessary toxicity for the low risk patients, and a high cost treatment.

MapQuant-Genome grade index (GGI), a molecular tool, was created to address this issue, and assess whether gene expression profiling could be used to grade tumor more accurately [12].

Development of the GGI was based on the evaluation of gene expression profile patterns obtained by microarray analysis of tumor specimens from a total 661 primary breast cancer. Histologic tumor grade was based on the Elston-Ellis grading system. Histologic grade 1 and 3 breast cancer were found to have distinct gene expression profiles, but histologic grade 2 tumors

had heterogeneous gene expression profiles that ranged from those for histologic grade 1 tumors to those for histologic grade 3. This observation led to the conclusion that the three-category histology grading system could be replaced with a two-category gene expression grading system. In multivariate analysis a stronger association was seen between relapse-free survival and the genomic grade index compared to relapse-free survival and histologic grade [12].

The importance of GGI was further corroborated by its ability to distinguish ER positive breast cancer in two distinct molecular subgroups. In a population of 666 ER positive samples, high GGI subgroup had worse overall survival compared to low GGI. The 10-year estimated expected rate of developing metastases increased with the increasing value of the GGI score, indicating its high discriminating value [17]. More recently, in a study of 229 patients treated with neoadjuvant chemotherapy (T/FAC - paclitaxel, fluorouracil, doxorubicin, cyclophosphamide), GGI scores were significantly ($p<0.04$) higher in patients with pathologic complete response. High GGI could also predict response to neoadjuvant chemotherapy independently of the hormone receptor status [18].

Based on these data, the use of GGI in daily practice should eliminate the variability inherent to tumor grade assessment, with greater impact in the subset of grade II breast cancer. GGI should also improve the performance of algorithms commonly used to assist physicians in the decision making process; eliminating the variability in the tumor grade assessment has the potential to positively influence the prognostic and predictive powers of Adjuvant On Line (AOL) and the Nottingham Prognostic Index (NPI).

This trial aims to evaluate the feasibility of implementing the Genomic grade index in specialised breast cancer centers. The primary objective of this trial is to show that the MapQuant Dx™ Genomic Grade can be obtained in at least 80% of the patients, in a routine clinical practice setting.

2. Objectives:

2.1 Primary objective:

- To assess feasibility of implementing GGI in the subset of node negative and 1-3 node positive breast cancer in clinical practice.

2.2 Secondary evaluations - descriptive analysis:

- Baseline tumor and patient characteristics (age, pathologic tumor size, histologic grade, lymph nodes invasion, ER, PR, HER2 status and patient co-morbidities), genomic grade index, and patient classification with commonly used risk classification systems (AOL and NPI)

- Comparison between genomic grade index and histologic grade, ER, PR and HER2 status assessed by histologic and genomic methods

- Treatment recommendations according to commonly used risk classification systems AOL and Nottingham prognostic index (NPI) and genomic grade index.

- Treatment recommendations according to AOL, NPI (calculated with the classic histologic grade) and NPI and AOL (calculated with GGI result)

3.0 Eligibility Criteria

- Histologically confirmed invasive breast cancer meeting the following criteria:

    - Invasive breast cancer.

    - T1, T2, or operable T3 disease

    - Zero to three positive lymph nodes and no distant metastases

    - Unilateral tumor

        - Multifocal tumors are allowed provided that samples from each tumors are provided for GGI quantification
        - Ductal carcinoma in situ or lobular carcinoma in situ allowed if invasive cancer is present

- Operable disease
    - Must have undergone breast-conserving surgery or mastectomy with either a sentinel node procedure or full axillary clearance
- No other invasive cancer within the past 5 years except for adequately treated carcinoma in situ of the cervix or non melanoma skin cancer
- No psychological, familial, sociological, or geographical condition that would preclude entering into a clinical study

4.0 Study design

The objective of the proposed trial is to assess the feasibility of implementing a molecular tool (MapQuant Dx^{TM} Genomic Grade) in clinical daily practice. To participate in the study hospitals must have multidisciplinary breast cancer care structures that use standard operating procedures and have at least one dedicated physician (surgeon, pathologist, or medical oncologist) as a local coordinator. Assessing GGI requires working on fresh tissue, which is not a standard collection procedure in all centres; therefore patient's permission and informed consent before surgery will be required. Immediately after surgery a fresh tumor sample will be taken from the surgical piece, and put on RNA preservative to be sent for analysis (see appendix 1 for detailed description). The interval between surgery and the outpatient consultation should be maintained according to each institution policy. After pathological examination of the tumor tissue, the treating physician will fill-out the 1st section of the case report form (CRF) with

patient and tumor characteristics, the initial treatment recommendation, and the treatment recommendations if the GGI score would be low or high. The filled CRF section 1 will be sent to Insitut Jules Bordet. The treating physician will then fill CRF section 2 containining genome grade index result, and the final treatment decision after GGI result and discussion with the patient.. The final treatment decision after GGI result and discussion with the patient refers to the treatment that is finally prescribed. This last part of the study should not happen later than 15 working days after receiving the GGI result. No treatment strategy is imposed per protocol, and the final decision will be taken as usual by the treating physician after discussion with the patient.

There is no planned follow-up period. The trial will end after all the information from the accrued patients is received at Institute Jules Bordet.

Investigators will be informed on recruitment closure.

4.1 Study design summary

- Informed Consent obtained
- Fresh biopsy collected at time of breast cancer surgery to be sent in RNA preservative (RNA later) to DNA vision.
- CRF section 1: Initial treatment recommendation and treatment recommendations based on a theoretical GGI result (GGI low or high) – Fax it to Institut Jules Bordet
- The MapQuant GGI result will be sent to the participating center after the first CRF page is received at Institut Jules Bordet, within five working days.
- CRF section 2: Genomic grade index result and final treatment decision after GGI result and discussion with the patient. Fax it to Institut Jules Bordet up to 15 working days after receiving the GGI result.

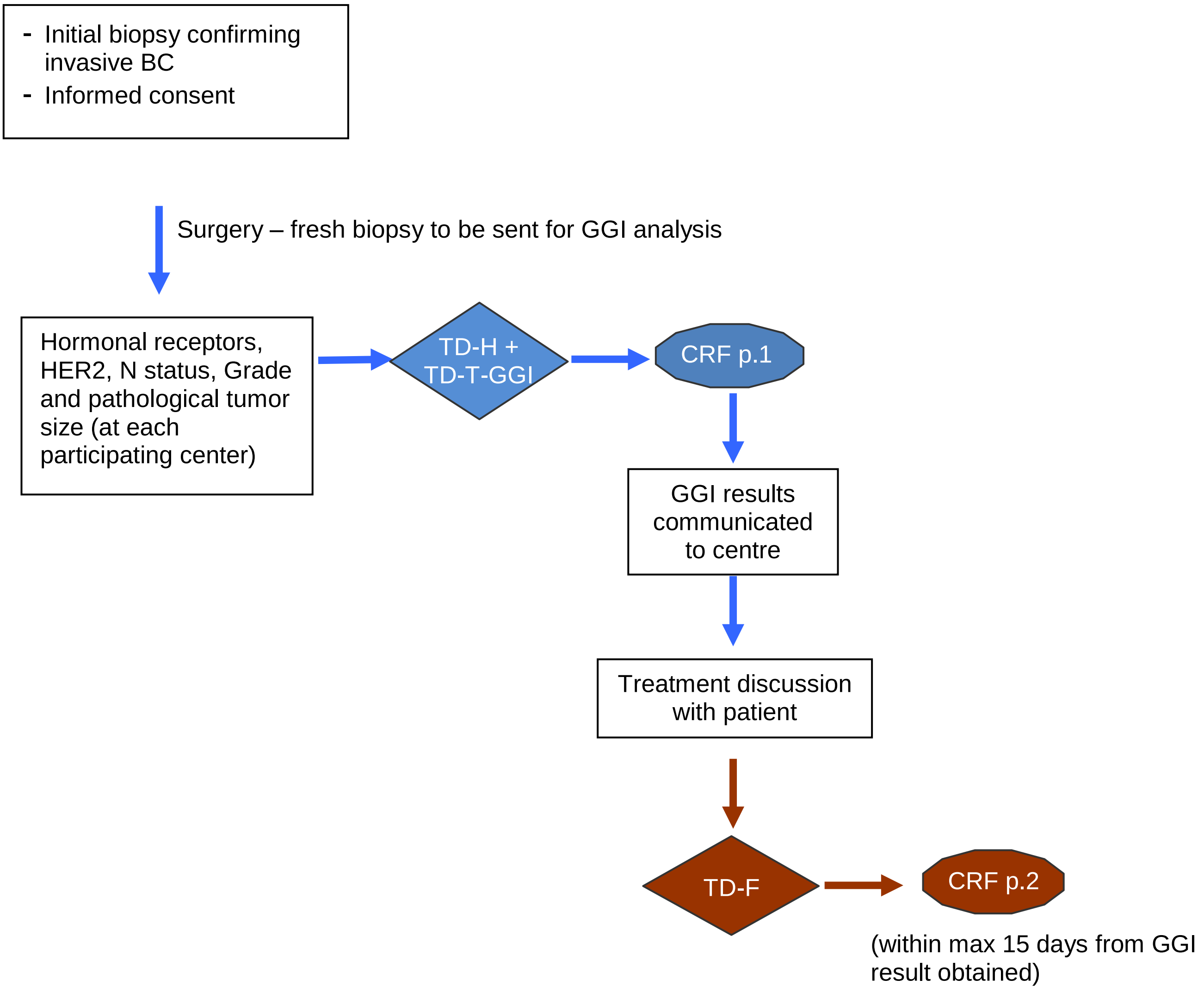

TD-H and TD-T-GGI: Treatment Decisions (H=Histology and T=Theoretical, if GGI would be low or high) made by treating centre consensus conference.
TD-F: Treatment Decision Final: made with the patient when all information is available for discussion.

5.0 Statistical considerations

Sample size calculation

The main objective of this trial is to show that the MapQuant Dx^{TM} (Genomic Grade index) can be obtained in at least 70% of the patients, in a routine clinical practice setting. The trial will test the following one-sample hypotheses on the "success" rate (*i.e.* a genomic grade is obtained):

$$H_0: p \leq 0.70 \quad \text{vs.} \quad H_A: p \geq 0.80$$

The sample size is calculated to have a power of 90% at a one-sided significance level of 5%.

Based on these hypotheses, the trial will require 137 patients (using an empirical estimate of the variance).

Descriptive statistics

- Baseline tumor and patient characteristics (age, tumor size, histologic grade, lymph nodes, ER, PR, HER2 status and patient co-morbidities), genomic grade index, and commonly used risk classification systems (Adjuvant! and Nottingham prognostic index).

- Concordance between genomic grade index and histologic grade, and between ER, PR and HER2 status determined by histology and genomic methods.

- Treatment recommendations according to commonly used risk classification systems AOL and Nottingham prognostic index (NPI) and genomic grade index.

- Treatment recommendations according to AOL, NPI (calculated with the classic histologic grade) and NPI and AOL (calculated with GGI result)

- To describe treatment recommendations in the subset of histological grade II breast cancer patients according to baseline characteristics (age, tumor size, lymph nodes, ER, PR, HER2 status and patient co-morbidities), genomic grade index, and commonly used risk classification systems (Adjuvant! and Nottingham prognostic index)

6.0 Timelines

- Expected study duration – 12 months in each center.
- The study will be closed after the sample size is obtained. It is not necessary to have a balanced accrual between participant centers.

7.0 Participating centers and principal investigators (PI)

**1**	**Hôpitaux Iris Sud Site-Ixelles (CHEI)**<br>Rue Jean Pacquot, 63<br>1050 Bruxelles	Docteur Jean-Pierre Haibe-Kains<br>jpkains@gmail.be<br>jpkains@his.irisnet.be<br>0475/87.73.24
**2**	**Centre Hospitalier Universitaire Tivoli**<br>Avenue Max Buset, 34<br>7100 La Louvière	Nathalie CORNEZ<br>Nathalie.Cornez@chu.tivoli.be<br>064/27.73.31<br>064/27.74.16
**3**	**CHU Ambroise Paré**	Vincent Richard<br>vincent.richard@hap.be<br>065/39.37.39
**4**	**Clinique et Maternité Sainte-Elisabeth**<br>Place Louise Godin, 15<br>5000 Namur	Dr Peter VUYLSTEKE<br>Peter.Vuylsteke@cmsenamur.be<br>081/72.04.11
**5**	**Grand Hôpital de Charleroi**<br>**Site Notre Dame**<br>Grand'Rue, 3<br>6000 Charleroi	Docteur J-L CANON<br>Canon.JL@ghdc.be<br>071/10.47.00
**6**	**RHMS – Clinique Louis Caty Baudour**<br>Rue Louis Caty, 136<br>7331 Baudour	Brigitte VANDERSCHUREN<br>brigitte.vanderschuren@rhms.be<br>0477/60.51.82
**7**	**CH St Joseph**	Pino Cusumano<br>pino@cusumano.be<br>043685299
**8**	**CHU de Liège**	Pino Cusumano<br>pino@cusumano.be<br>043685299

References:

1. *American Cancer Society Breast Cancer Facts and Figures 2007-2008 and Surveillance Research* 2007.
2. Peto, R., et al., *UK and USA breast cancer deaths down 25% in year 2000 at ages 20-69 years.* Lancet, 2000. **355**(9217): p. 1822.
3. Perou, C.M., et al., *Molecular portraits of human breast tumours.* Nature, 2000. **406**(6797): p. 747-52.
4. Sorlie, T., et al., *Gene expression patterns of breast carcinomas distinguish tumor subclasses with clinical implications.* Proc Natl Acad Sci U S A, 2001. **98**(19): p. 10869-74.
5. Sotiriou, C., et al., *Breast cancer classification and prognosis based on gene expression profiles from a population-based study.* Proc Natl Acad Sci U S A, 2003. **100**(18): p. 10393-8.
6. Hu, Z., et al., *The molecular portraits of breast tumors are conserved across microarray platforms.* BMC Genomics, 2006. **7**: p. 96.
7. Sotiriou, C. and L. Pusztai, *Gene-expression signatures in breast cancer.* N Engl J Med, 2009. **360**(8): p. 790-800.
8. Haibe-Kains, B., et al., *Comparison of prognostic gene expression signatures for breast cancer.* BMC Genomics, 2008. **9**: p. 394.
9. *Effects of chemotherapy and hormonal therapy for early breast cancer on recurrence and 15-year survival: an overview of the randomised trials.* Lancet, 2005. **365**(9472): p. 1687-717.
10. *Endocrine responsiveness and tailoring adjuvant therapy for postmenopausal lymph node-negative breast cancer: a randomized trial.* J Natl Cancer Inst, 2002. **94**(14): p. 1054-65.
11. Berry, D.A., et al., *Estrogen-receptor status and outcomes of modern chemotherapy for patients with node-positive breast cancer.* Jama, 2006. **295**(14): p. 1658-67.
12. Sotiriou, C., et al., *Gene expression profiling in breast cancer: understanding the molecular basis of histologic grade to improve prognosis.* J Natl Cancer Inst, 2006. **98**(4): p. 262-72.
13. Elston, C.W. and I.O. Ellis, *Pathological prognostic factors in breast cancer. I. The value of histological grade in breast cancer: experience from a large study with long-term follow-up.* Histopathology, 1991. **19**(5): p. 403-10.

14. Fitzgibbons, P.L., et al., *Prognostic factors in breast cancer. College of American Pathologists Consensus Statement 1999.* Arch Pathol Lab Med, 2000. **124**(7): p. 966-78.

15. Singletary, S.E., et al., *Revision of the American Joint Committee on Cancer staging system for breast cancer.* J Clin Oncol, 2002. **20**(17): p. 3628-36.

16. Paik, S., et al., *Supplement: A multigene assay to predict recurrence of tamoxifen-treated, node-negative breast cancer.* N Engl J Med, 2004. **351**(27): p. 2817-26.

17. Loi, S., et al., *Definition of clinically distinct molecular subtypes in estrogen receptor-positive breast carcinomas through genomic grade.* J Clin Oncol, 2007. **25**(10): p. 1239-46.

18. W. F. Symmans, C.H., C. Liedtke, C. Desmedt, V. Valero, H. M. Kuerer, G. N. Hortobagyi, M. Piccart-Gebhart, L. Pusztai, C. Sotiriou, *Use of genomic grade index (GGI) to predict pathologic response to preoperative chemotherapy in breast cancer.* J Clin Oncol 2008 **26**(May 20 suppl; abstr 541).

APPENDIX 1: technical description of tissue sampling and processing for a GGI analysis, using the Map*Quant* Dx Path Kit

Introduction

Context

Sample collection procedures, transport and storage conditions are crucial to analyze gene expression levels in a sample. RNA is very labile and degradation can occur immediately after tissue resection. It is therefore very important to prevent RNA degradation by stabilizing RNA as quickly as possible after tissue harvesting, and to avoid further degradation by storing and shipping the sample at adequate temperature conditions.

Preservative solution in the kit preserves RNA 1 day at 37°C, 7 days at +15 / +25°C or 3 weeks at 4°C. A temperature indicator ensures that the sample has not been exposed to temperatures that degrade its RNA.

Indications on use and Performance

Kit for tissue collection and RNA preservation during storage and transport.

Preservative solution in the kit preserves RNA 1 day at 37°C, 7 days at +15 / +25°C or 3 weeks at 4°C.

Equipment to be supplied by the User

1. Disposable gloves to prevent RNA degradation by RNases present on the hands
2. If possible, paper towel or aluminium to cover surfaces while manipulating tissue samples

Safety information

The material safety data sheet of the RNA preservative solution is available on request.

Handle the sample with care when cutting it with sharp tools.

The human tissue must be handled as if potentially infectious and should be removed with special precautions, in agreement with the EU-OSHA - the European Agency for Safety and Health at Work.

Never pipette the reagents by mouth and avoid contact with skin and mucous membranes. If reagent is exposed to sensitive areas, wash thoroughly with water and contact a physician.

- Time between tissue resection and sample handling should be reduced at the minimum (ideally 15 minutes, max 1 hour), in order to prevent gene expression changes and RNA degradation.

- This kit can be used for fresh or frozen tissue only, but not for fixed tissue. See sampling procedure below.

- This kit is for single use only. Do not reuse.

- Do not dilute or substitute tissue collection solution with any other solution as it may result in a loss of preserving capacity and the product may chemically react dangerously with some agents.

Sampling and shipping procedure

- **Verify that the kit is still valid** (check the expiry date indicated on the back of the box).
- **Wear gloves throughout the procedure when manipulating tissues.**

- **Fresh and frozen samples should at least weight 25mg and must be representative of the tissue to be analyzed.**

1. Fresh sample collection

The sample must be taken on surgically resected tissue within one hour.

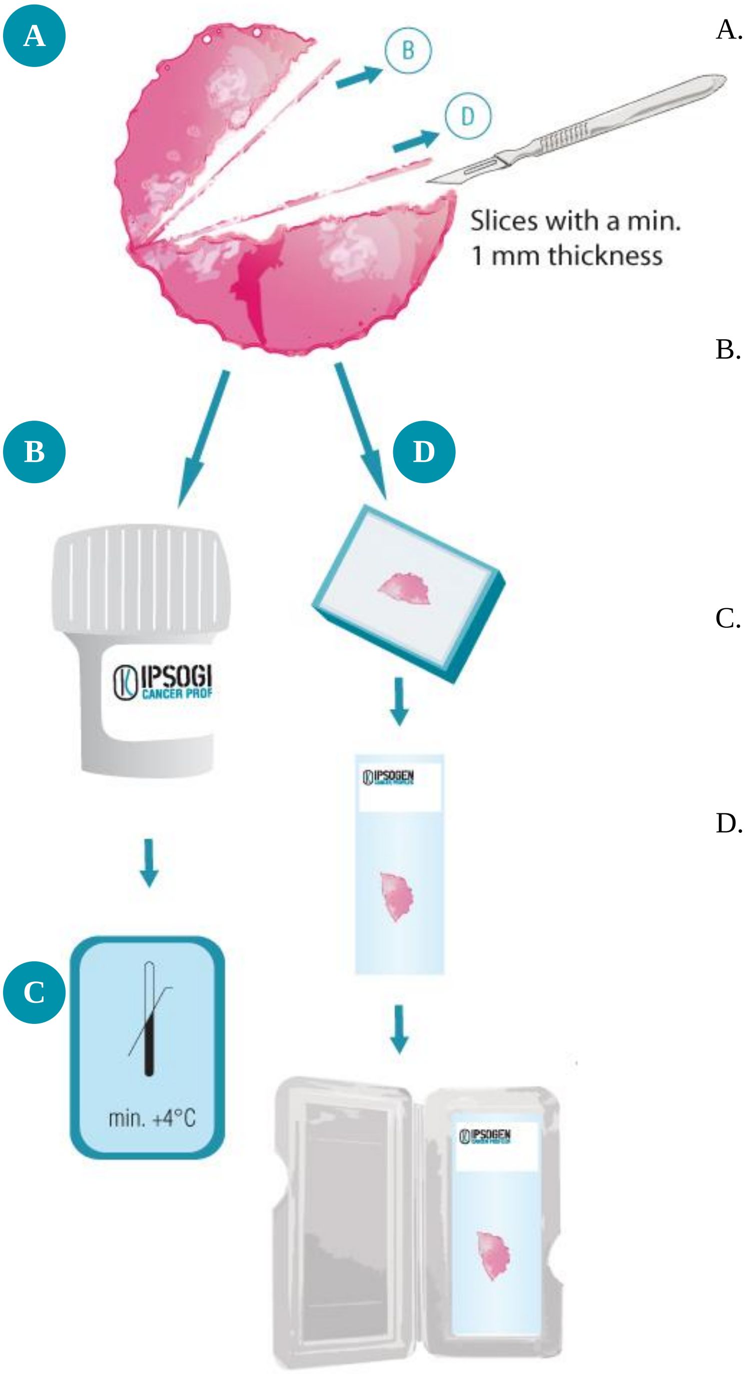

A. Sample a slice of tissue with at least 1 mm-thickness on a section of 25mm² (5 mm on side or 6 mm diameter). Use preferably the scalpel supplied in the kit or, if not, any other disposable device.

B. Immediately immerse the tissue slice obtained in the preservative solution contained in the tube so that the reagent covers the entire sample.

C. Store the tube containing the sample at ambient temperature or at 4°C before shipment to the laboratory.

D. Provide a histological slide from the adjacent tissue section of the sample so that the cell content of the sample can be evaluated. We recommend Haematoxylin Eosin Safran (HES) staining.

2. Sample shipment to the Laboratory

Once collected, the sample should be immediately immersed in the solution for the preservation and the RNA stabilization contained in the tube.

The tube containing the sample should be sent within the 3 days to the laboratory.

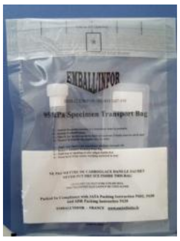

A. Put the tube in the 95kPa IATA compliant pouch, supplied with the kit.

B. Securely close the bag following the instructions indicated on it.

C. Place the histological slide in the dedicated mailer box supplied.

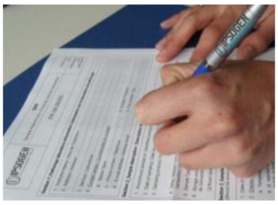

D. Complete the Sample Report Form (SRF).

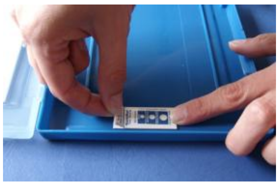

E. Activate the temperature indicator by pulling the plastic protective film. From that point until delivery, the temperature indicator will monitor any temperatures over 37°C.

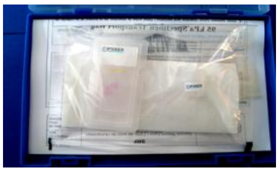

F. Fold the bag and place it in the outer plastic box with the histological slide mailer box and the SRF.

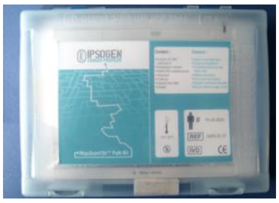

G. Close the box with the security seal label (supplied).

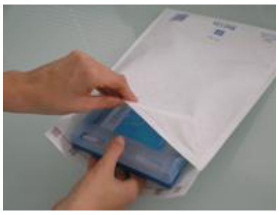

H. Ship the box as soon as possible at room temperature to DNAVision SA (25, avenue Georges Lemaitre B-6041 Gosselies-Charleroi, Belgium.

Tel: + 32 71 37 85 27 / Fax: + 32 71 37 85 01).

Contact: Jean-François Laes (Head of the microarray unit)

CAUTION: Ship at ambient temperature.

Do not freeze sample or use dry ice for shipment.
