# Supplementary material for: Genomic Grade Index (GGI): Feasibility in Routine Practice and Impact on Treatment Decisions in Early Breast Cancer
Source: PLoS One. 2013 Aug 19;8(8):e66848. doi: 10.1371/journal.pone.0066848 (PMC3747186; doi:10.1371/journal.pone.0066848)
Supplement: Text S1 — MapQuant Dx Process. (PDF) [file pone.0066848.s004.pdf]

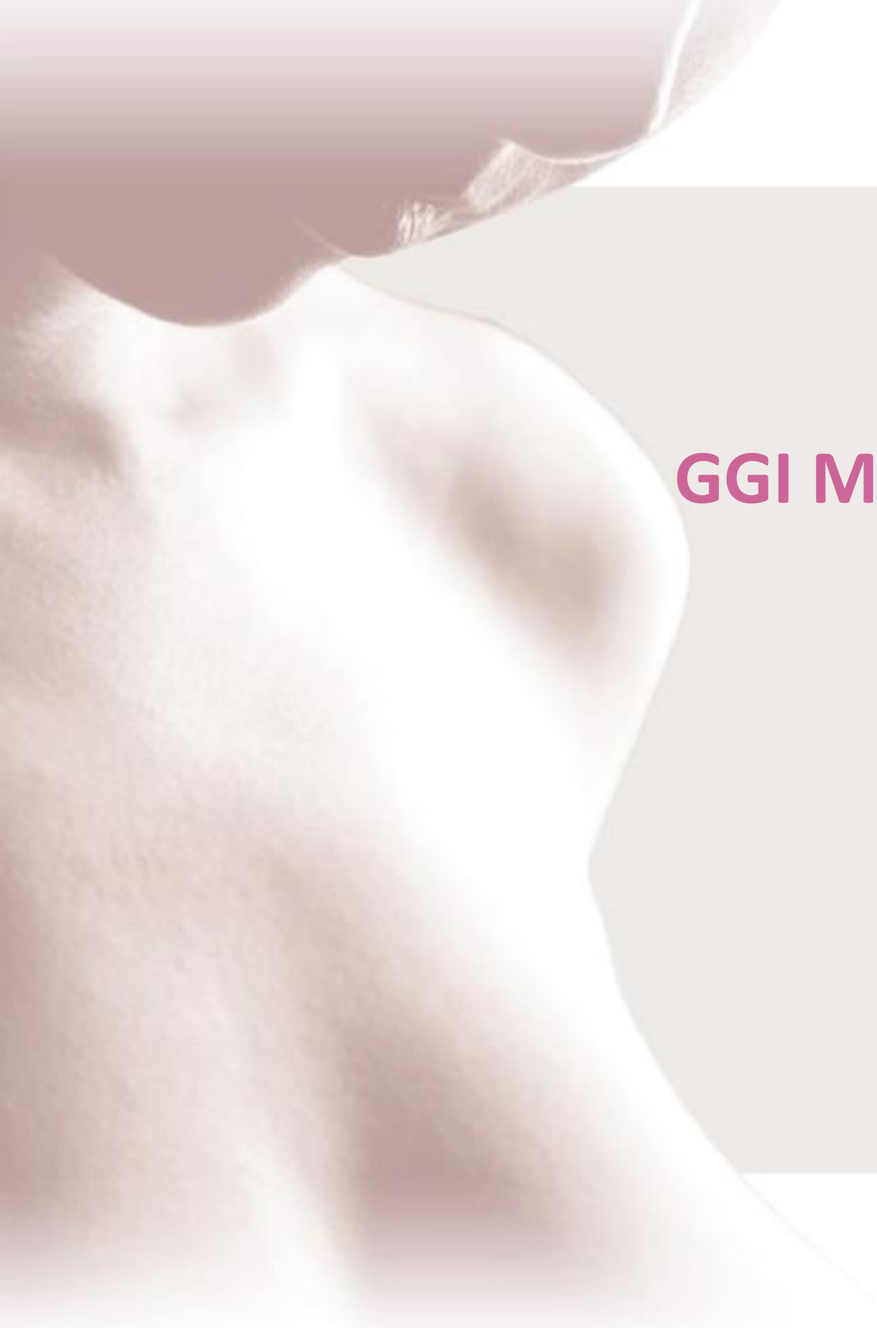

GGI Map*Quant* Dx™ Process

Feasability Study

IJB, Brussels 26/10/2009.

Map*Quant* Dx™ Process

A 3-step process

PATHOLOGY LABORATORY → MOL BIOL LABORATORY → BIOINFO

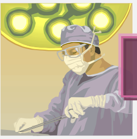

Tumor Sampling

Conditioning/Shipping

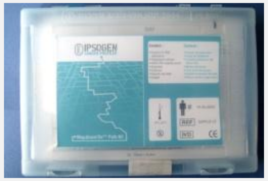

Map*Quant* Dx Path Kit

Sample Processing & Profiling

DNAVISION YOUR GENETIC SOLUTION ISO 17025

- QCs
- Grinding
- RNA Extraction
- Hybridization
- Pangenomic Profile

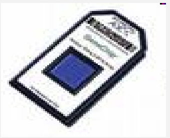

AFFYMETRIX

GGI computing

IPSOGEN CANCER PROFILER

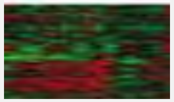

Map*Quant Dx* ™ Software

- QC
- Calibration

Map*Quant* Dx™ GG Test Report @

10 days from Path Kit reception

IPSOGEN PROFILING BREAST CANCER

Tumour Sampling Process

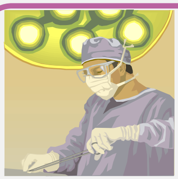

Surgical resection

SURGEON

Fresh tumour - No fixative

1 hour Max

Immediate transport
to pathology department

PATHOLOGIST

Sampling : MapQuant Dx Path Kit
Immediate immersion in RNA preserving solution
- Room Temperature or 4°C -

Shipping @ RT:
reception at DNAV lab within 7 days

IPSOGEN
PROFILING BREAST CANCER

Tumour Sampling from fresh tissue and Storage

Map*Quant* Dx™ Path Kit: tissue sample collection & RNA preservation during storage and transport

PATHOLOGY LABORATORY

Sampling

- Surgical biopsy
- representative sample
- Ø 3 mm min.
- **Fresh**

Immediate immersion in preservative solution (1 hour max after resection)

Storage

- **never freeeze**
- **+4°C or RT**

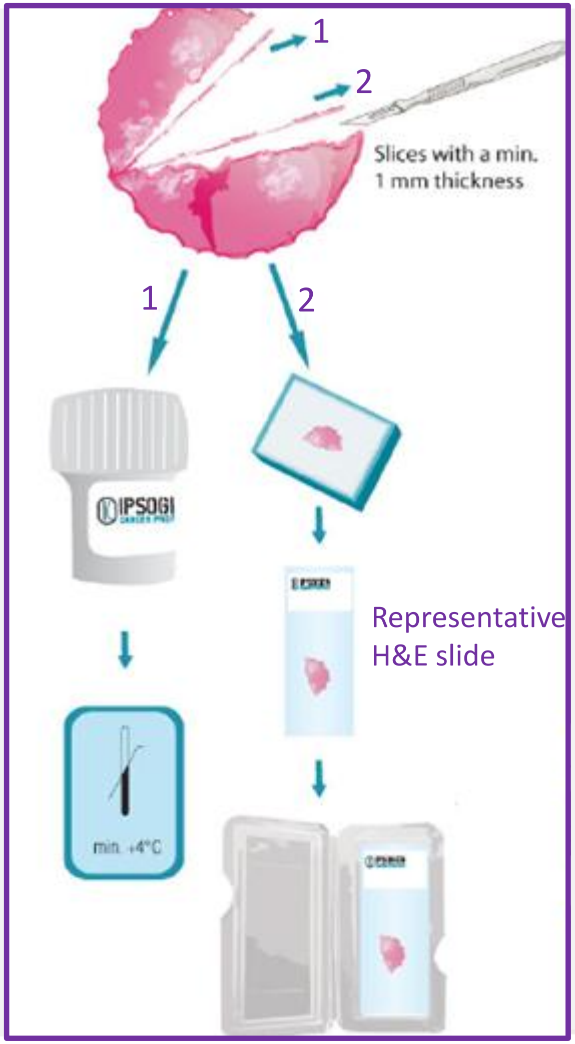

IPSOGEN
PROFILING BREAST CANCER

Tumour Sampling from frozen tissue and Storage

MapQuant Dx™ Path Kit: tissue sample collection & RNA preservation during storage and transport

Nerver defrost

Frozen tissue

200µm (20µm microtome slices x10)

Immediate immersion in preservative solution
(1 hour max after resection)

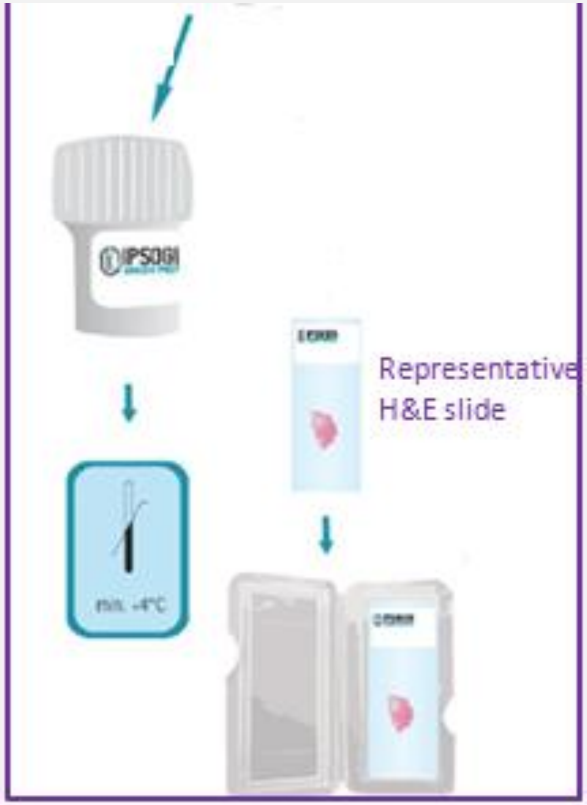

Storage

- never freeeze
- +4°C or RT

IPSOGEN
PROFILING BREAST CANCER

Map*Quant* Dx Path Kit preparation for shipping

SRF

IPSOGEN PROFILING BREAST CANCER	SRF<br>Sample Report Form / Fiche de suivi de l'échantillon	FQP01-02<br>V02
	PK-09-XXXXX	Effective as of/au 14/04/2009

Section 1_Pathology laboratory information / Information sur le laboratoire de pathologie

1. Institution Name / *Nom de l'Institution*:
2. Address / *Adresse*:
3. Service / *Laboratoire*:
4. Pathologist details / *Coordonnées de l'Anatomopathologiste*:
   a. Name / *Nom*: b. Position / *Fonction*:
   c. Phone number / *Téléphone*: d. Fax number / *Fax*:
   e. E-mail address / *Adresse email*:

Section 2_Sample description / Description de l'échantillon

1. Sample Id / Identifiant de l'échantillon: ……………………
2. Date of sampling / Date de collecte: |__|__| / |__|__| / |__|__|__|__|
3. Sampling protocol / Protocole de collecte: ❑ As recommended / Comme recommandé (cf. IFU/ notice) ❑ Other / Autre: ……………………………………
4. Time between tumor resection and sampling / Délai entre la résection de la tumeur et la collecte de l'échantillon (HH/MM): _ _ /_ _
5. Sample / Echantillon: ❑ Fresh tissue / Tissu frais ❑ Frozen tissue / Tissu congelé
6. Histological grade / Grade histologique : ❑ Grade 1 ❑ Grade 2 ❑ Grade 3
7. Percentage of tumor cell / Cellularité tumorale > 30%: ❑ Yes / Oui ❑ No / Non ❑ ND
8. Storage conditions in the preservative solution before shipment / Conditions de stockage dans la solution de préservation avant transport:
   ❑ Room temperature / Température ambiante (HH/MM) ❑ +4°C Duration (HH/MM): _ _/_ _

Section 3_Sample Shipment / Expédition de l'échantillon
(Should be done within three days post storage in preservative solution / *Doit être réalisé dans les trois jours après stockage dans la solution de préservation*)

1. Date of shipment / *Date de l'expédition*: |__|__| / |__|__| / |__|__|__|__|
2. Checks / Contrôles :
   a. Temperature tag was activated / *La sonde de température a été activée* ❑
   b. Id reported on tube, slide, SRF and box are identical / *Les identifiants reportés sur le tube, la lame, la fiche de suivi et la boîte sont les mêmes* ❑
   c. HE/S control slide included / *Lame contrôle HE/S incluse* ❑

Once completed, place this form, slide mailer and bag containing tube & absorbent in the outer blue box. Close the box and seal it with the security seal label.

Placer ce formulaire rempli ainsi que le porte-lame et le sachet contenant le tube & la matière absorbante dans la boîte bleue. Fermer la boîte puis sceller avec l'étiquette de sécurité.

Je déclare avoir pris connaissance et avoir compris les conditions générales d'utilisation du test MapQuant Dx™ Genomic Grade / I acknowledged that I have read and understood the general conditions of use of the MapQuant Dx™ Genomic Grade (www.ipsogen.com)

______________________________ date and signature

Path Kit

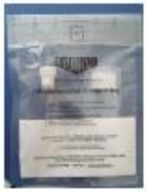

- Specimen in RNA preservative solution
- H&E slide

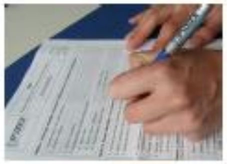

- SRF

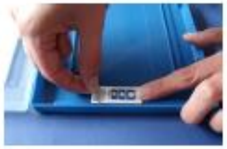

- Temperature probe

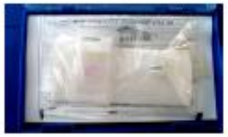

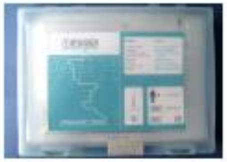

- IATA 650 compliant packaging

Shipping ASAP

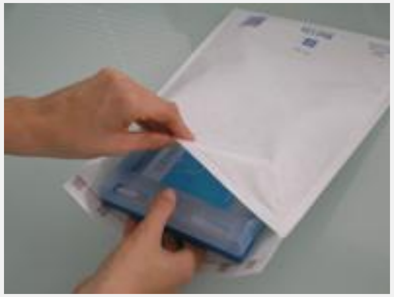

+15°C to +25°C
(NO DRY ICE)

IPSOGEN PROFILING BREAST CANCER

Section 1_Pathology laboratory information / Information sur le laboratoire de pathologie

1. Institution Name / *Nom de l'Institution*:
2. Address / *Adresse*:
3. Service / *Laboratoire*:
4. Pathologist details / *Coordonnées de l'Anatomopathologiste*:
   a. Name / *Nom*:
   b. Position / *Fonction*:
   c. Phone number / *Téléphone*:
   d. Fax number / *Fax:*
   e. E-mail address / *Adresse email*:

Section 2_Sample description / Description de l'échantillon

1. Sample Id / Identifiant de l'échantillon: ………………………
2. Date of sampling / Date de collecte: |__|__| / |__|__| / |__|__|__|__|
3. Sampling protocol / Protocole de collecte: ❑ As recommended / Comme recommandé (cf. IFU/ notice)
   ❑ Other / Autre: ……………………………………………
4. Time between tumor resection and sampling / Délai entre la résection de la tumeur et la collecte de l'échantillon (HH/MM): _ _ /_ _
5. Sample / Echantillon: ❑ Fresh tissue / Tissu frais ❑ Frozen tissue / Tissu congelé
6. Histological grade / Grade histologique : ❑ Grade 1 ❑ Grade 2 ❑ Grade 3
7. Percentage of tumor cell / Cellularité tumorale > 30%: ❑ Yes / Oui ❑ No / Non ❑ ND
8. Storage conditions in the preservative solution before shipment / Conditions de stockage dans la solution de préservation avant transport:
   ❑ Room temperature / Température ambiante (HH/MM) ❑ +4°C Duration (HH/MM): _ _/_ _

Section 3_Sample Shipment / Expédition de l'échantillon

(Should be done within three days post storage in preservative solution / *Doit être réalisé dans les trois jours après stockage dans la solution de préservation*)

1. Date of shipment / *Date de l'expédition*: |__|__| / |__|__| / |__|__|__|__|
2. Checks / Contrôles :
   a. Temperature tag was activated / *La sonde de température a été activée* ❑
   b. Id reported on tube, slide, SRF and box are identical / *Les identifiants reportés sur le tube, la lame, la fiche de suivi et la boîte sont les mêmes* ❑
   c. HE/S control slide included / *Lame contrôle HE/S incluse* ❑

Once completed, place this form, slide mailer and bag containing tube & absorbent in the outer blue box. Close the box and seal it with the security seal label.

Placer ce formulaire rempli ainsi que le porte-lame et le sachet contenant le tube & la matière absorbante dans la boîte bleue. Fermer la boîte puis sceller avec l'étiquette de sécurité.

Je déclare avoir pris connaissance et avoir compris les conditions générales d'utilisation du test Map*Quant* Dx™ Genomic Grade / I acknowledged that I have read and understood the general conditions of use of the Map*Quant* Dx™ Genomic Grade (www.ipsogen.com)

____

date and signature

MapQuant Dx Genomic Grade Test Report

- Test report sent by e-mail and FAX:

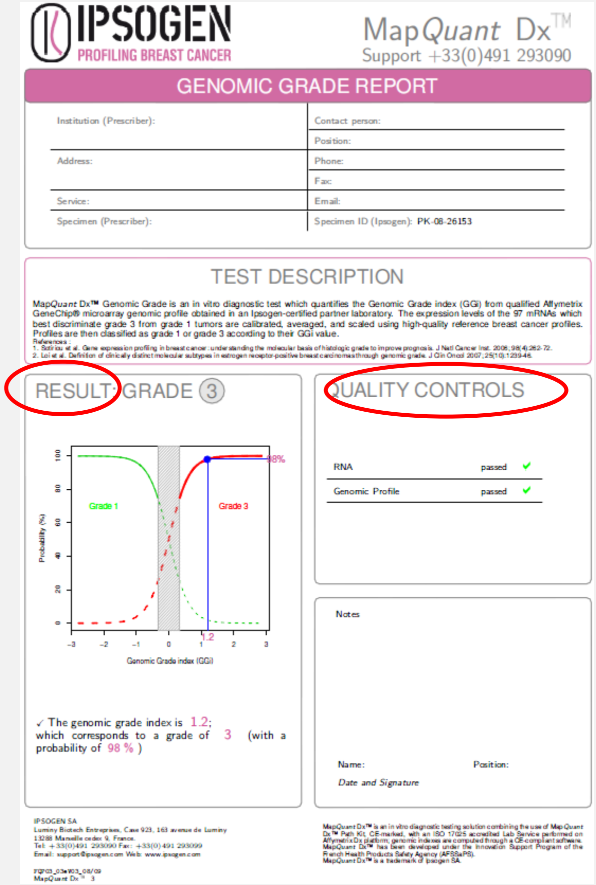

IPSOGEN
PROFILING BREAST CANCER

MapQuant Dx™
Support +33(0)491 293090

GENOMIC GRADE REPORT

Institution (Prescriber):	Contact person:
	Position:
Address:	Phone:
	Fax:
Service:	Email:
Specimen (Prescriber):	Specimen ID (Ipsogen): PK-08-26153

TEST DESCRIPTION

Map*Quant* Dx™ Genomic Grade is an in vitro diagnostic test which quantifies the Genomic Grade index (GGi) from qualified Affymetrix GeneChip® microarray genomic profile obtained in an Ipsogen-certified partner laboratory. The expression levels of the 97 mRNAs which best discriminate grade 3 from grade 1 tumors are calibrated, averaged, and scaled using high-quality reference breast cancer profiles. Profiles are then classified as grade 1 or grade 3 according to their GGi value.

References :
1. Sotiriou et al. Gene expression profiling in breast cancer: understanding the molecular basis of histologic grade to improve prognosis. J Natl Cancer Inst. 2006;98(4):262-72.
2. Loi et al. Definition of clinically distinct molecular subtypes in estrogen receptor-positive breast carcinomas through genomic grade. J Clin Oncol 2007;25(10):1239-46.

RESULT: GRADE 3

✓ The genomic grade index is 1.2; which corresponds to a grade of 3 (with a probability of 98 %)

QUALITY CONTROLS

RNA	passed	✓
Genomic Profile	passed	✓

Notes

Name: Position:

Date and Signature

IPSOGEN SA
Luminy Biotech Entreprises, Case 923, 163 avenue de Luminy
13288 Marseille cedex 9, France.
Tel: +33(0)491 293090 Fax: +33(0)491 293099
Email: support@ipsogen.com Web: www.ipsogen.com

FQP03_03eV03_08/09
MapQuant Dx™ 3

MapQuant Dx™ is an in vitro diagnostic testing solution combining the use of MapQuant Dx™ Path Kit, CE-marked, with an ISO 17025 accredited Lab Service performed on Affymetrix Dx platform; genomic indexes are computed through a CE-compliant software. MapQuant Dx™ has been developed under the Innovation Support Program of the French Health Products Safety Agency (AFSSaPS).
MapQuant Dx™ is a trademark of Ipsogen SA.

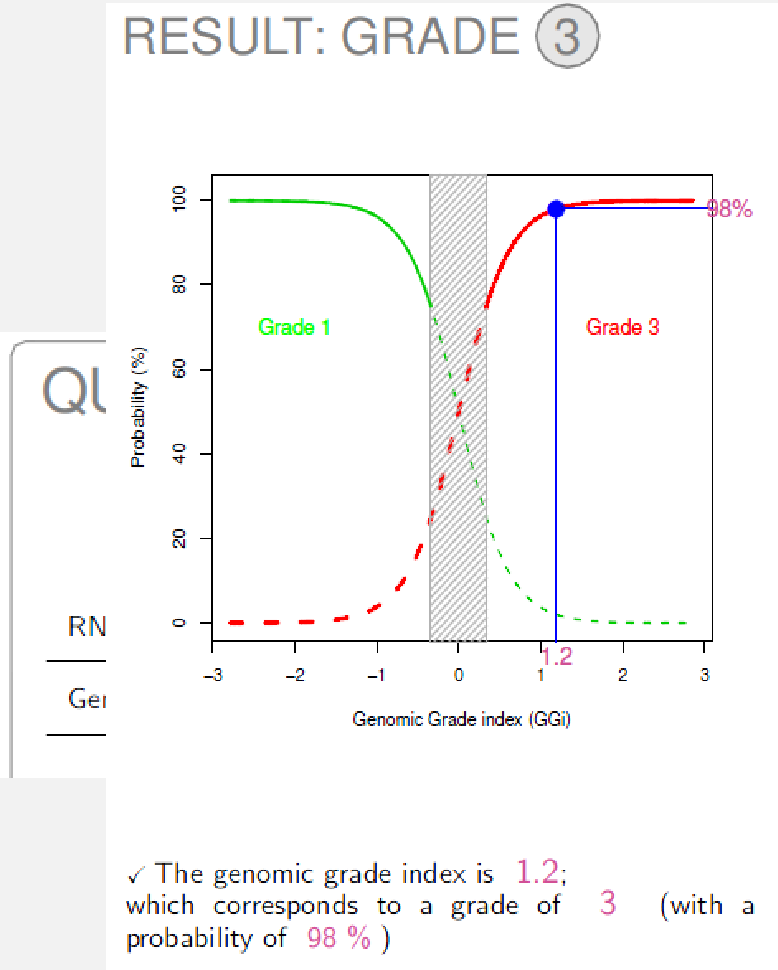

IPSOGEN
PROFILING BREAST CANCER
