# Supplementary material for: Genomic Grade Index (GGI): Feasibility in Routine Practice and Impact on Treatment Decisions in Early Breast Cancer
Source: PLoS One. 2013 Aug 19;8(8):e66848. doi: 10.1371/journal.pone.0066848 (PMC3747186; doi:10.1371/journal.pone.0066848)
Supplement: Text S2 — Central Ethics Committee Approval. (PDF) [file pone.0066848.s005.pdf]

Institut Jules Bordet

Association Hospitalière de Bruxelles
Centre des Tumeurs de l'ULB
Association régie par la loi du 8 juillet 1976
Rue Héger-Bordet, 1 - 1000 BRUXELLES
Tél : 02 541 31 11
DEXIA 091-0097278-16

Comité d'Ethique

Tél : 02/541.35.95
Fax : 02/541.37.95
Secrétariat email :
Comite.ethique@bordet.be

Président
Dr. Th. Gil

Vice-Président
Dr. D. Bron

Secrétaire
Dr. D. Lossignol

Membres
Dr. H. Bleiberg
Dr. E. de Azambuja
Dr. J. Klastersky
Dr. M. Sosnowski
Dr. Th. Renard
Monsieur P. Crombez
(suppléante : Madame B. Fernez)
Mme M. Colin
Mme M. Paesmans
Mme L. Van Ingelgem
M. R. Nuyts

# LIST OF DOCUMENTS REVIEWED BY THE MEDICAL ETHICS COMMITTEE OF INSTITUT JULES BORDET - LEC

**Réf. : as**

**Date : 18/02/2010**

**Eudract : 2009-015521-36**

**Intern number : 1668**

**Coordinating investigator : Dr. SOTIRIOU**

**Principal investigators : Doctors Machiels (Cliniques Universitaires St Luc), Cornez (CHU Tivoli), Neven (KUL), Vanderschuren (RHMS – Baudour), Canon (Grand Hôpital de Charleroi – Site Notre Dame), Vuylsteke (Clinique St Elisabeth à Namur) et Haibe – Kains (Hôpitaux Iris Sud - Site Ixelles)**

**Title**
**MapQuant DxTM Genomic Grade: feasibility in routine practice and impact of tumor grade quantification on treatment decision-making in early breast cancer patients**

**Including**

- CTApplication form
- Protocole version 4.0 dd 17/02/2010 incorporating Appendix 1
- Synopsis
- ICF version 4.0 dd 17/02/2010 (français et néerlandais)
- Genome grade index : feasability and impact on treatment decision
- Certificat d'assurance dd 21/08/2009

*1668 suite (18/02/2010)*

| |
|---|
| **LEADING ETHICS COMMITTEE'S DECISION** |
| **X Approval** |
| **☐ Approval with conditions** |
| **☐ Need for extra information** |
| **☐ Disapproval** |

| |
|---|
| Signature of the Chairman of the Central Ethics Committee |
| ........................... |
| Name : Dr. Th. GIL |
| Date : 18/02/2010 |
